# Supplementary material for: Referential equations for pulmonary diffusing capacity using GAMLSS models derived from Japanese individuals with near-normal lung function
Source: PLoS One. 2022 Jul 21;17(7):e0271129. doi: 10.1371/journal.pone.0271129 (PMC9302824; doi:10.1371/journal.pone.0271129)
Supplement: S1 File — (DOCX) [file pone.0271129.s001.docx]

**APPENDIX:
Instruction for using the D_LCO_ excel sheet and examples of calculating the predictive values**

**1. Instruction for using the D_LCO_ excel sheet**

1. To calculate each predicted value using an Excel sheet, you must alter the blue columns for each subject (age [years], height [cm], D_LCO_ [ml/min/mmHg], T_LCO_ [mmol/min/kPa], K_CO_ [ml/min/mmHg/L], and V_A_ [L] ).
2. Do not directly enter numbers into each yellow column, which displays the results.

**2. Examples of calculating the predictive values**

Herein, we illustrate the calculation of the predictive pulmonary function values (denoted by$\hat{\mu}$), the lower limit of normal (LLN), and *Z*-scores from the best GAMLSS model. We consider the following examples for calculating the D_LCO_ values for men:

**1.1 Calculating the predicted D_LCO_ value for men aged 65 years, with a height of 170 cm**

From Table 4, we obtained the following best GAMLSS model for the D_LCO_ values in men:

$$\ln\left( \mu\right)=-3.05697+1.42677\times\ln\left( \mathrm{height} \right)-0.26486\times\ln\left( \mathrm{age} \right)$$

$$+M-spline$$

$$\ln\left( \sigma\right)=-3.1864+0.2488\times\ln\left( \mathrm{age} \right)+S-spline$$

$$\nu=-2.4$$

with the D_LCO_ in ml/min/mmHg, age in years, and height in cm.
Therefore, the D_LCO_ (ml/min/mmHg) value for a 65-year-old man with a height of 170 cm was calculated using the median model as follows:

$\hat{\mu}=\exp\left\{ -3.0567+1.42677\times\ln\left( 170 \right)-0.26486\times\ln\left( \mathrm{age} \right)+0.001025475 \right\}=23.713$

$$\sigma=\exp\left\{ -3.1864+0.2488\times\ln\left( 65 \right)+0.00000285 \right\}=0.11674$$

**1.2 Calculating the LLN of the D_LCO_ value for men aged 65 years, with a height of 170 cm**

The LLN is obtained using the equation postulated by Rigby, Stasinopoulos (2004) [1]and Cole (1992) [2], i.e., $lower limit of normal \left[ 5th percentile \right]:\exp\left[ \ln\left[ \hat{\mu} \right]+\frac{\ln\left[ 1-1.645\cdot L\cdot S \right]}{L} \right]$where $z_{0.05}=-1.645$ for BCCG distribution.

Therefore, for men aged 65 years, with a height of 170 cm,
the LLN of D_LCO_=$\exp\left[ \ln\left[ 23.713 \right]+\frac{\ln\left[ 1-1.645\cdot-2.4\cdot0.11674 \right]}{-2.4} \right]=20.249$

**1.3 Calculating the *Z*-score for a man with observed D_LCO_ 23.00 ml/min/mmHg, aged 65 years, with a height of 170 cm**

The *Z*-score was obtained using the Equation (1) postulated by Rigby, Stasinopoulos (2004) and Cole(1992) as follows:

$Z=\frac{1}{\sigma\nu}\times\left\{ \left( \frac{Y}{\mu} \right)^{\nu}-1 \right\}.$

Therefore, the Z-score for a man with the observed D_LCO_ of 23.00 ml/min/mmHg, aged 65 years, with a height 170 cm was as follows:

$Z=\frac{1}{0.11674\times(-2.4)}\times\left\{ \left( \frac{23.0}{23.713} \right)^{-2.4}-1 \right\}=20.249$.

Supplementary figure caption

Fig 1. Bland-Altman plots depicting differences in the mean predicted lung indices between current values, the Global Lung Function Initiative 2017 reference values, and those of Nishida et al. and Burrows et al. in men and women.


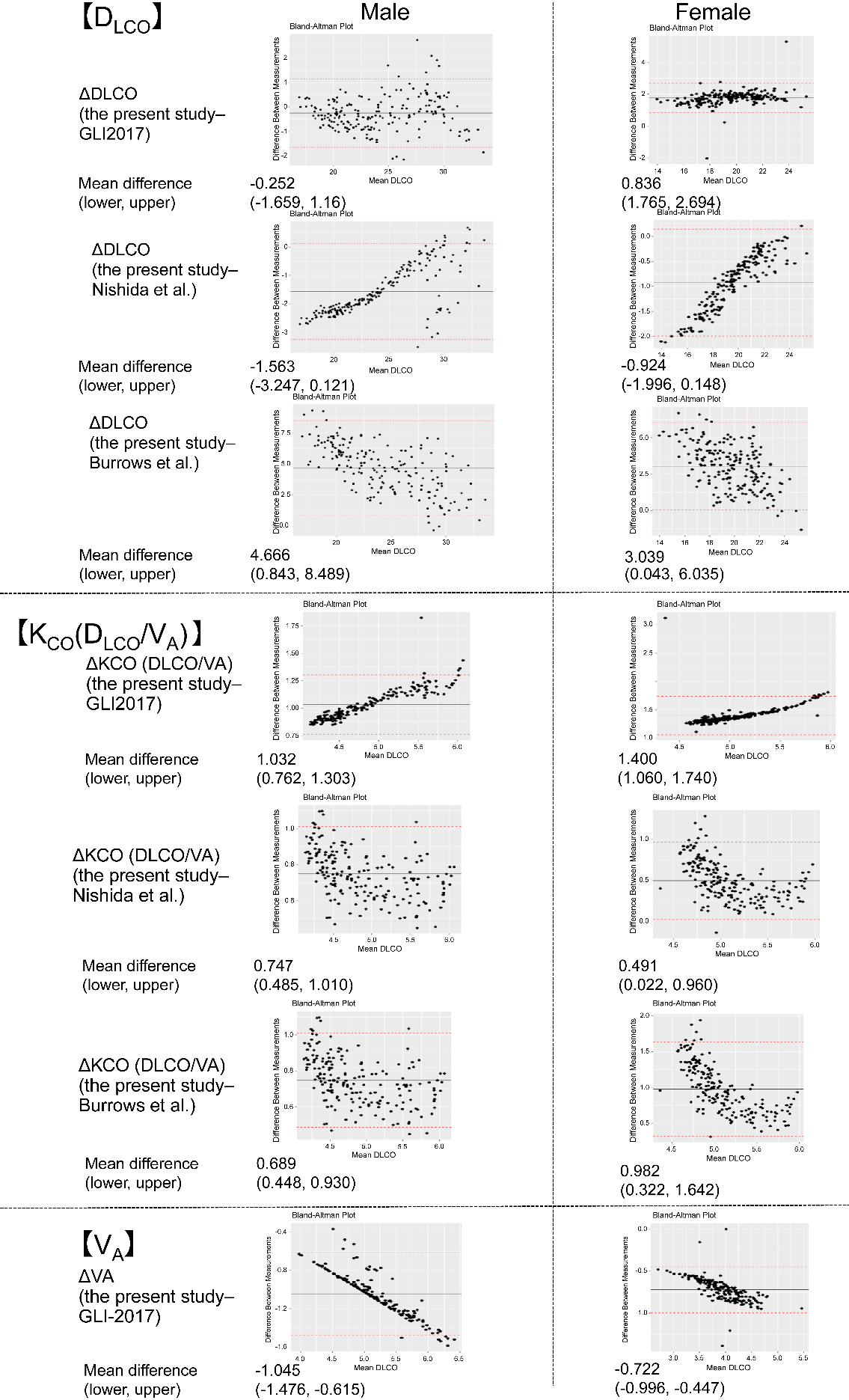


Abbreviations: D_LCO_: single-breath diffusing capacity for carbon monoxide; K_CO_: single breath diffusing capacity for carbon monoxide per unit of lung volume; V_A_: alveolar volume; GLI-2017: Global Lung Function Initiative 2017 reference values [2]; Nishida et al.: Nishida et al. reference values [13]; and Burrows et al.: Burrows et al. reference values [14].

**Supplementary Reference**

1. Rigby RA, Stasinopoulos DM. Smooth centile curves for skew and kurtotic data modelled using the Box-Cox power exponential distribution. *Stat Med* 2004: 23(19): 3053-3076.

2. Cole TJ, Green PJ. Smoothing reference centile curves: the LMS method and penalized likelihood. Stat Med. 1992;11: 1305-1319.
